# A 3D-Printed Sensor for Monitoring Biosignals in Small Animals

**DOI:** 10.1155/2017/9053764

**Published:** 2017-10-25

**Authors:** Sung-Joon Cho, Donghak Byun, Tai-Seung Nam, Seok-Yong Choi, Byung-Geun Lee, Myeong-Kyu Kim, Sohee Kim

**Affiliations:** ^1^School of Electrical Engineering and Computer Science, Gwangju Institute of Science and Technology (GIST), Gwangju 61005, Republic of Korea; ^2^School of Mechanical Engineering, Gwangju Institute of Science and Technology (GIST), Gwangju 61005, Republic of Korea; ^3^Chonnam National University Medical School, Gwangju 61469, Republic of Korea; ^4^Department of Robotics Engineering, Daegu Gyeongbuk Institute of Science and Technology (DGIST), Daegu 42988, Republic of Korea

## Abstract

Although additive manufacturing technologies, also known as 3D printing, were first introduced in the 1980s, they have recently gained remarkable popularity owing to decreased costs. 3D printing has already emerged as a viable technology in many industries; in particular, it is a good replacement for microfabrication technology. Microfabrication technology usually requires expensive clean room equipment and skilled engineers; however, 3D printing can reduce both cost and time dramatically. Although 3D printing technology has started to emerge into microfabrication manufacturing and medical applications, it is typically limited to creating mechanical structures such as hip prosthesis or dental implants. There have been increased interests in wearable devices and the critical part of such wearable devices is the sensing part to detect biosignals noninvasively. In this paper, we have built a 3D-printed sensor that can measure electroencephalogram and electrocardiogram from zebrafish. Despite measuring biosignals noninvasively from zebrafish has been known to be difficult due to that it is an underwater creature, we were able to successfully obtain electrophysiological information using the 3D-printed sensor. This 3D printing technique can accelerate the development of simple noninvasive sensors using affordable equipment and provide an economical solution to physiologists who are unfamiliar with complicated microfabrication techniques.

## 1. Introduction

Although classical microfabrication technologies have allowed for miniaturization of devices in broad fields adapted from the integrated circuit (IC) to nanoelectromechanical systems (NEMS), it is limited in building complex three-dimensional (3D) geometrical structures. There have been many attempts to develop techniques to reduce complicated fabrication steps but to build complex 3D structures [1, 2]. These microfabrication approaches however still require specialized high-cost equipment and skilled engineers. This remains a large barrier for scientists who need microdevices in their research but are unfamiliar with microfabrication techniques. Recently, additive manufacturing techniques, better known as 3D printing, have arisen interest as a key technology in the manufacturing industry and have been heralded as a third industrial revolution [3]. 3D printers have enabled rapid structuring of versatile shapes with minimal infrastructure by simply converting computer-aided design (CAD) files into a physical object by fusing plastics, metals, ceramics, powders, or even living cells [4, 5]. With these advantages, 3D printing technologies have started to be used frequently in consumer sectors such as the medical industry, food industry, and fashion industry [6–8], and their market value is expected to reach US$16.2 billion by 2018 [9].

Biomedical applications and healthcare devices have also gained significant interest, but medical applications of 3D printing techniques have been limited to mainly the creation of structural prosthetics for implantation and tissue engineering for transplantation [10–13]. A sensor refers to any device that acquires a stimulus from an object of interest and returns with an electrical signal [14]. Obtaining accurate information from physical measurements is crucial in diagnosis. Especially, measuring and quantifying such electrical activity provides fundamental information of abnormal rhythms during electrophysiological studies toward a means for monitoring health status [15]. A sensor plays a big role in detecting the electrical activities; therefore, a sensor can determine the quality of electrophysiological results.

Although there was an attempt to manufacture dry EEG electrodes using 3D printing technique, implementation of classical sensor fabrication techniques was not achieved which limits the use of the method in the fabrication of smaller objects [16]. 3D printing can provide a customized product with an increased cost efficiency and productivity. In the development of noninvasive healthcare devices, high-sensitivity sensors are critical to the measurement of electrophysiological signals, such as provided by electroencephalograms (EEG) and electrocardiograms (ECG) [17]. However, sensor technology using 3D printing has not made much progress because classical sensor fabrication techniques are not compatible with plastics, which are the most affordable and common materials used in 3D printing because plastics are vulnerable to chemical and heat degradations [18]. Among bioelectric signals such as EEG, ECG, electromyogram (EMG), and electrooculogram (EOG), EEG and ECG can tell about the two most important life-related body parts—namely, the brain and the heart. While the amplitudes of EMG and EOG signals are in the millivolt range, the amplitudes of EEG and ECG are in the microvolt range. Therefore, EEG and ECG require more sensitive sensors due to the smaller amplitudes of the signals when compared to other bioelectric signals.

The zebrafish is an animal model that is increasingly used in physiological studies. It is becoming more popular due to its cost effectiveness, genetic accessibility, experimental accessibility, and generation of large numbers of offspring [19–21]. However, due to its small body size and demand for the wet condition, physiological studies using zebrafish have mainly relied on invasive methods or behavioral tests [20, 22, 23].

In this study, we propose a 3D-printed sensor that can monitor multiple types of bioelectric signals, which is cost-effective and time-efficient in fabrication. The performance of the sensor was examined by measuring biosignals noninvasively from the zebrafish. By using the 3D-printed sensor, we were able to overcome the aforementioned conventional problems in zebrafish electrophysiology studies. The sensitivity of the sensor was verified by successfully measuring the EEG and ECG signals in the microvolt range. The selectivity of the sensor was confirmed by successfully measuring multichannel EEG from the zebrafish, which has a cranial area of only 2 mm × 2 mm.

## 2. Materials and Methods

The sensing area of the electrode was sized to be 300 *μ*m wide in order to fit the cranial area of the zebrafish, which is approximately 2 mm × 2 mm. The electrodes were printed using a 3D printer (Ultimaker 2, Ultimaker BN, Geldermalsen, The Netherlands). The nozzle temperature was set to 200°C, and rafts and supporting materials were not used to print the electrodes. Polylactic acid (PLA) filaments were chosen to print the electrodes with an extrusion speed of 30 mm/s on the bed, where the temperature was set to 50°C.

After the printing process was complete, Ti/Au was deposited onto the printed electrodes to provide the proper electrical characteristics as a bioelectric sensor. 50 nm of Ti was deposited to serve as an adhesion layer, and then 100 nm of Au was deposited using an electron beam evaporator (REP5004, SNTEK, Suwon-si, South Korea). Then, wires were attached to the object using a conductive epoxy (DURALCO 125, Cotronics Corp., New York, USA) for electrical connection to the signal acquisition device.

**Figure 1:**
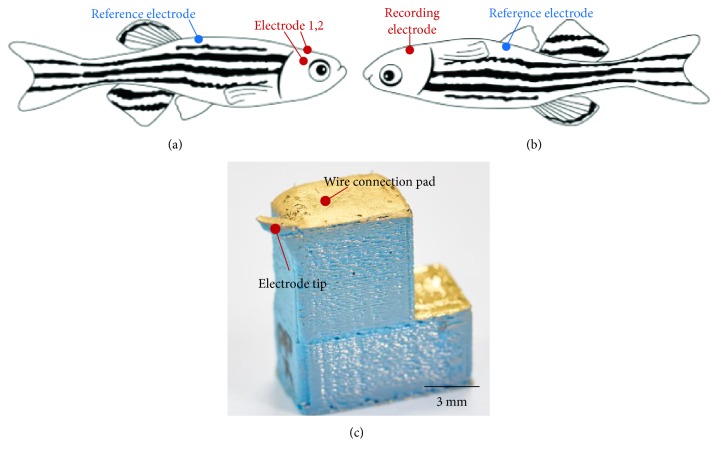
(a, b) 3D-printed electrode placement schemes. Tips of two recording electrodes were placed on the dorsal part of the zebrafish for EEG recording while a recording electrode was placed on the ventral part for ECG recording. Reference electrodes were placed on supraneural spine and belly for EEG and ECG recordings, respectively. (c) Photograph of the 3D-printed bioelectric sensor.

After the wire was connected to the electrodes, parylene C was selectively deposited onto the electrodes to electrically insulate it using a chemical vapor deposition system (PDS 2010, Specialty Coating Systems, Indiana, USA), except for the tip. The tip of the electrodes, in a diameter of about 300 *μ*m, was masked using a dental impression material (PERFECT-F VPS, Han Dae Chemical Co., Jinchun-kun, South Korea) to keep the region from the deposition of the insulation material. By using the dental impression material, the electrodes were selectively insulated and no damage occurred to the deposited metal after all the fabrication processes were complete, and no deformation of the printed electrodes was found.  Figure 1(c) shows a picture of the completed electrodes.

Wild-type adult zebrafish were purchased and maintained under a 14/10 hr light/dark cycle. Animal experiments were approved by the Institutional Animal Care and Use Committee of the Gwangju Institute of Science and Technology. Prior to recording, the zebrafish were anesthetized with 15 ppm clove oil extract (Eugenol E51791, Sigma-Aldrich, Missouri, USA) as described in [19].

To ensure that the 3D-printed sensors were capable of being used as electrophysiological sensors, EEG and ECG were measured from the zebrafish. The wires from each electrode were connected to the signal acquisition system (MP36, BIOPAC Systems Inc., California, USA). For both EEG and ECG recordings, band-pass filters were applied from 0.1 Hz to 50 Hz to eliminate DC components and 60 Hz noise, and the gain was set to 10 K.  Figure 1 shows the electrode placements for both types of recordings. During the recording sessions, zebrafish were supported using clay to prevent losing balance.

For EEG recording, a total of three printed electrodes were used. Two electrodes were placed on each hemisphere of the zebrafish cranium, and the other electrode was used as a reference electrode that was placed on the supraneural spines (Figure 1(a)), the most stable place for reference electrode positioning during EEG recording [19, 21]. To detect brain responses, we measured event-related potentials (ERP) while a photic stimulation was applied repetitively. To record ECG signals from the zebrafish, the zebrafish was placed upside down, and one electrode was placed in the anterior position as a recording electrode, while the other electrode was placed in the posterior position as a reference electrode (Figure 1(b)).

Both EEG and ECG measurements were performed on five animals (*n* = 5) each, at room temperature (25°C) for up to three minutes. All recordings were monitored using the biosignal acquisition hardware (MP36, BIOPAC Systems Inc., California, USA) and were displayed and saved on a Biopac Student Lab 4.1 software (BIOPAC Systems Inc., California, USA). Fast Fourier transform (FFT) analysis was performed to display the power spectrum of the EEG data in the frequency domain. The recorded raw data were postprocessed in order to analyze the signal morphology and to obtain more accurate ECG waveform parameters. A peak detection algorithm was used to detect and sort the R peaks from the recorded raw ECG signals. All waveforms from a minute gap-free signals within 30% difference in amplitude and duration that contained the same ECG shapes were matched together [24, 25]. Then, the obtained waveforms were averaged.

## 3. Results and Discussion

Figure 1(c) shows the 3D-printed electrode. Metals were successfully deposited on the PLA surface without causing deformation. Adhesion between the printed electrode and the deposited Ti/Au was sufficiently strong such that no damage was found after the wire connection and parylene C deposition processes.

**Figure 2:**
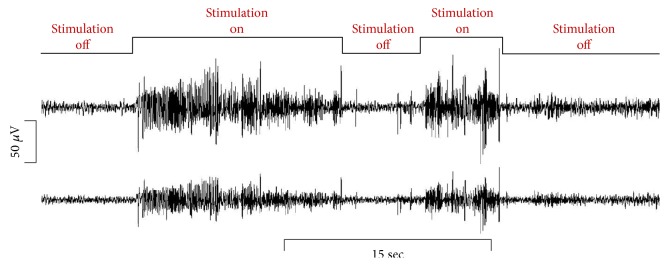
Recorded raw EEG signals for 45 seconds from two channels of the 3D-printed sensor. When photic stimulation was given, amplitudes were significantly increased.

**Figure 3:**
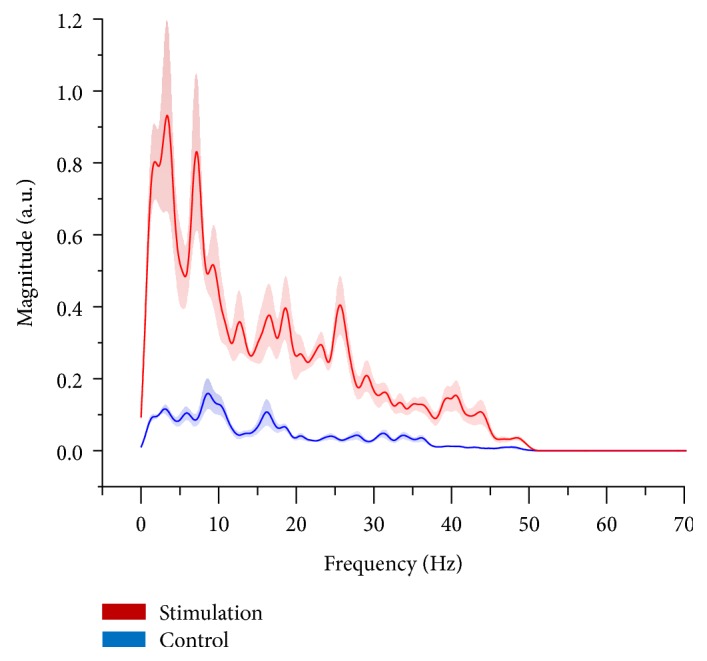
Averaged FFT power spectra for the photic stimulated state and the control state. Solid lines depict the means and shaded areas depict the standard deviations (red: photic stimulation group; blue: control group).

Figure 2 shows the recorded two-channel EEG signals from the zebrafish. During the photic stimulation, the amplitudes of the signal were significantly increased compared to the normal state when there was no stimulation.  Figure 3 represents the averaged FFT results from the five zebrafish. During the normal stage, activity in the alpha band (8–15 Hz) was dominant. The alpha band typically indicates the relaxed state, which in this case corresponds to the fact that the zebrafish were under anesthesia. The beta band (16–31 Hz) and the gamma band (>32 Hz) showed almost no activity during the normal state. However, when the photic stimulation was on, higher frequency activities were significantly increased compared to the normal state. The beta band is known to be activated when the individual is stressed or in a high-alert state, and the gamma band is usually observed during sensory processing, such as that of sound and sight [26, 27].

**Figure 4:**
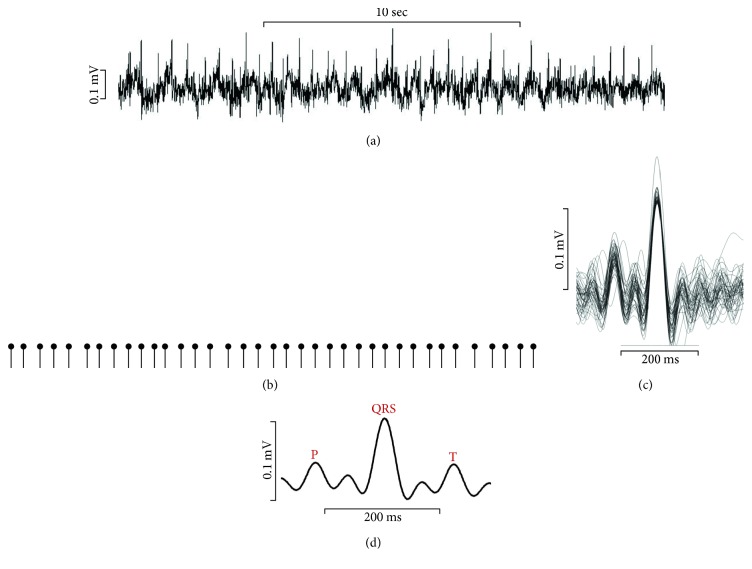
Recorded raw ECG signals and processed signals. (a) Representative ECG waveform for 20 seconds. (b) Identification and extraction of each heartbeat by R peak detection. (c) Overlay of extracted waveforms from 1-minute gap-free signal. (d) Averaged ECG waveform for signal morphology analysis.

Figure 4(a) represents the recorded ECG signals from the zebrafish. Using the 3D-printed sensor, constant ECG peaks were successfully detected. The morphology of the zebrafish ECG signals was very similar to that of human ECG signals. To study accurately the zebrafish ECG parameters, firstly, a peak detection algorithm was used to detect the R peaks from the recorded raw ECG signals (Figure 4(b)). This allowed easy calculation of the heart rate. The mean heart rate of the zebrafish was 133.8 ± 17.9 bpm (*n* = 5), which is consistent with previous studies on zebrafish heart rate [24, 25]. Then, the recorded ECG signals were overlaid (Figure 4(c)) and the individual waveforms were averaged (Figure 4(d)). The resultant ECG signals demonstrated all of the ECG components, P-wave, QRS-complex, and T-wave. The mean PR and QT intervals were 84.21 ± 11.98 ms and 221.31 ms ± 6.91 ms, respectively. The ECG parameters obtained using the 3D-printed sensor were within the deviation range of those previously reported [24, 25, 28].

## 4. Conclusions

A 3D-printed sensor that is capable of detecting bioelectric signals was introduced and demonstrated by measuring the EEG and ECG signals from zebrafish. The EEG and ECG signals were noninvasively measured from the animals using the developed sensor, which was built by economical and simple 3D printing methods. The 3D printing technique reduced both the cost and fabrication time significantly compared to traditional complicated microfabrication processes. Also, modifications of sensor designs are much easier using the 3D printing technique so that it allows for the fabrication of a greater variety of designs than were previously possible using microfabrication processes. We were able to fabricate a highly sensitive and selective sensor relying only on dry processes for the 3D-printed object, and we confirmed that the measured signals were from the brain and the heart of the animal. The parameters from the obtained signals were of the same nature as those previously reported. We were able to obtain the same electrophysiological information using the 3D-printed sensor as was measured using a sensor fabricated by more expensive methods. We believe that this technique can accelerate the development of noninvasive biomedical sensors and provide an economical solution to physiologists who are unfamiliar with complicated fabrication techniques.
